# Genomic and ecological study of two distinctive freshwater bacteriophages infecting a *Comamonadaceae* bacterium

**DOI:** 10.1038/s41598-018-26363-y

**Published:** 2018-05-22

**Authors:** Kira Moon, Ilnam Kang, Suhyun Kim, Sang-Jong Kim, Jang-Cheon Cho

**Affiliations:** 1 School of Biological Sciences, Seoul National University, Seoul, 08826 Republic of Korea; 2 Department of Biological Sciences, Inha University, Incheon, 22212 Republic of Korea

## Abstract

Bacteriophages of freshwater environments have not been well studied despite their numerical dominance and ecological importance. Currently, very few phages have been isolated for many abundant freshwater bacterial groups, especially for the family *Comamonadaceae* that is found ubiquitously in freshwater habitats. In this study, we report two novel phages, P26059A and P26059B, that were isolated from Lake Soyang in South Korea, and lytically infected bacterial strain IMCC26059, a member of the family *Comamonadaceae*. Morphological observations revealed that phages P26059A and P26059B belonged to the family *Siphoviridae* and *Podoviridae*, respectively. Of 12 bacterial strains tested, the two phages infected strain IMCC26059 only, showing a very narrow host range. The genomes of the two phages were different in length and highly distinct from each other with little sequence similarity. A comparison of the phage genome sequences and freshwater viral metagenomes showed that the phage populations represented by P26059A and P26059B exist in the environment with different distribution patterns. Presence of the phages in Lake Soyang and Lake Michigan also indicated a consistent lytic infection of the *Comamonadaceae* bacterium, which might control the population size of this bacterial group. Taken together, although the two phages shared a host strain, they showed completely distinctive characteristics from each other in morphological, genomic, and ecological analyses. Considering the abundance of the family *Comamonadaceae* in freshwater habitats and the rarity of phage isolates infecting this family, the two phages and their genomes in this study would be valuable resources for freshwater virus research.

## Introduction

Bacteriophages (phages) are the smallest but the most abundant biological entities on earth^1^. Bacteriophages mostly replicate through host bacterial cell lysis and hence, phages not only participate in bacterial population control but also intervene nutrient metabolism and cycling. Phages also have a role in controlling bacterial genetic diversity by mediating horizontal gene transfer through unintentional carriage of host gene fragments while infecting one host after the other^2^. In the process of carrying host genome fragments within the phage capsid, some phages acquire auxiliary metabolic genes (AMGs) within their own genome that can be expressed and alter the cell metabolism upon phage infection and eventually benefit phage reproduction. AMGs involved in photosystem, glycolysis, and phosphorous, sulfur, and nitrogen cycling have been found in many phage genomes and viral metagenomes^3–5^.

Despite the diverse functions of phages in the environment, available genomic resources of environmental phages are rather limited in quantity and diversity. One of the major constraints in phage studies is that respective bacterial hosts need to be cultured first to isolate their infecting phages. However, only a small proportion of bacteria in nature can be cultured in laboratory settings, and even if they are successfully cultured, maintaining those strains are challenging. In order to overcome the culturability restrictions, viral metagenome (virome) was suggested as an approach to discover the unexplored diversity of bacteriophage genomes^6^. Without the need of culturing both bacteria and phages, virome analysis provided access to large amounts of bacteriophage genomes in the environment. Through assembling virome reads, many studies reported novel and abundant bacteriophage genomic contigs in various environments^7–9^. These studies revealed that the analyses of genome (or genomic contig) sequences alone were not sufficient to provide information on the hosts or morphology of phages, which are the basic information required for phage classification. Although virome analyses disclosed large numbers of environmental viral sequences, the lack of independently isolated phages led to limited interpretation of the virome data. Therefore, virome approaches to study phages in the environment must be accompanied by the isolation and characterization of individual phages.

Most of the studies on environmental bacteriophages have been subjected for marine phages, isolating the most abundant bacteriophages such as pelagiphages and *Puniceispirillum* phages^10,11^, and surveying marine viral population through metagenome^8,12^. However, contrast to the marine environment, only a few studies have been performed in lakes, which include researches on freshwater cyanophages^13–16^. Only recently, the phages infecting the LD28 bacterial clade were cultured from an oligotrophic freshwater lake^17^, and metagenome-assembled genomes of the phages that are considered to infect the acI clade were retrieved from viromes^18^. Each lake across the globe is confined and independent from one another such that each has its own unique ecological values, regardless of their geographic locations and variation in climates and environments. Despite the large diversity in lakes, the major bacterial composition is very similar throughout lakes while the detailed community structure is different even in those under similar environmental conditions^19–21^, providing interesting aspects in freshwater microbial evolution and ecology. Thereby, extensive studies on individual freshwater bacterial groups have been performed by many researchers^22–24^. However, representatives of many abundant freshwater bacterial groups are still uncultured, along with their phages.

Lake Soyang, located in South Korea, is the largest artificial lake in Korea that is designated as a source water protection area. As a well conserved oligotrophic lake, Lake Soyang is inhabited by diverse bacterial lineages and phage groups. To study the microbial population dynamics of this lake, a number of bacterial strains and bacteriophages have been isolated including IMCC26059, a bacterial strain used as a host in this study. Strain IMCC26059 belongs to the family *Comamonadaceae* of the class *Betaproteobacteria*^25^. The family *Comamonadaceae* is one of the dominant bacterial groups in freshwater environments and includes well-known freshwater bacterial genera such as *Limnohabitans* and *Rhodoferax*^20^. Despite the abundance and ubiquity of *Comamonadaceae* in freshwater habitats, phages infecting members of this family were rarely studied in freshwater habitats. To our knowledge, only one lytic phage that infects a strain of *Rhodoferax*, and three phages that infect *Delftia* of the family *Comamonadaceae* have been reported so far^26,27^. In this study, to obtain more freshwater phages that infect *Comamonadaceae*, lytic bacteriophages have been screened from Lake Soyang using the IMCC26059 strain as a host, which resulted in the successful isolation of two phages, P26059A and P26059B. Morphological analysis and whole genome sequencing were performed for the two phages. Subsequently, the distribution of the two phages in diverse freshwater environments was determined using the genomes of the phages and freshwater virome data, contributing to the understanding of freshwater phage populations and their diverse genomes.

## Results and Discussion

### Isolation of two phages infecting the *Comamonadaceae* strain IMCC26059

Strain IMCC26059, the host strain of this study, was isolated from Lake Soyang. In a similarity analysis of the 16 S rRNA gene sequences using EzBioCloud^28^, IMCC26059 showed a close relationship with several type strains of the family *Comamonadaceae*: *Curvibacter delicatus* (97.93%), *Curvibacter fontanus* (97.92%), *Rhodoferax saidenbachensis* (97.85%), and *Albidiferax ferrireducens* (97.27%). A phylogenetic tree built based on 16 S rRNA gene sequences also placed IMCC26059 clearly within the family *Comamonadaceae*, although the taxonomic status within the family could not be resolved unambiguously (Supplementary Fig. S1).

**Figure 1:**
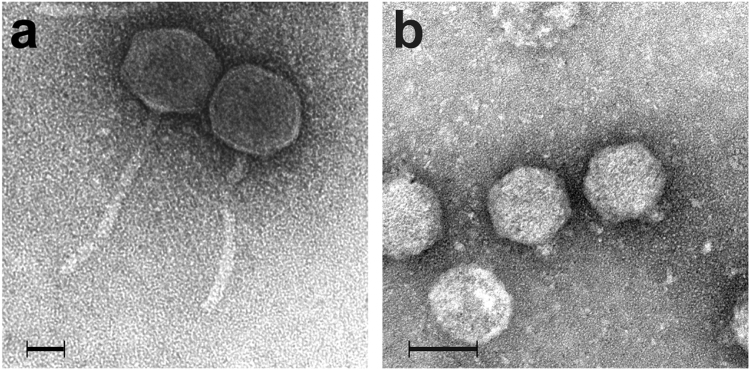
Transmission electron microscopic images showing the morphology of phages P26059A (**a**) and P26059B (**b**). The scale bars represent 20 nm (**a**) and 50 nm (**b**).

**Figure 2:**
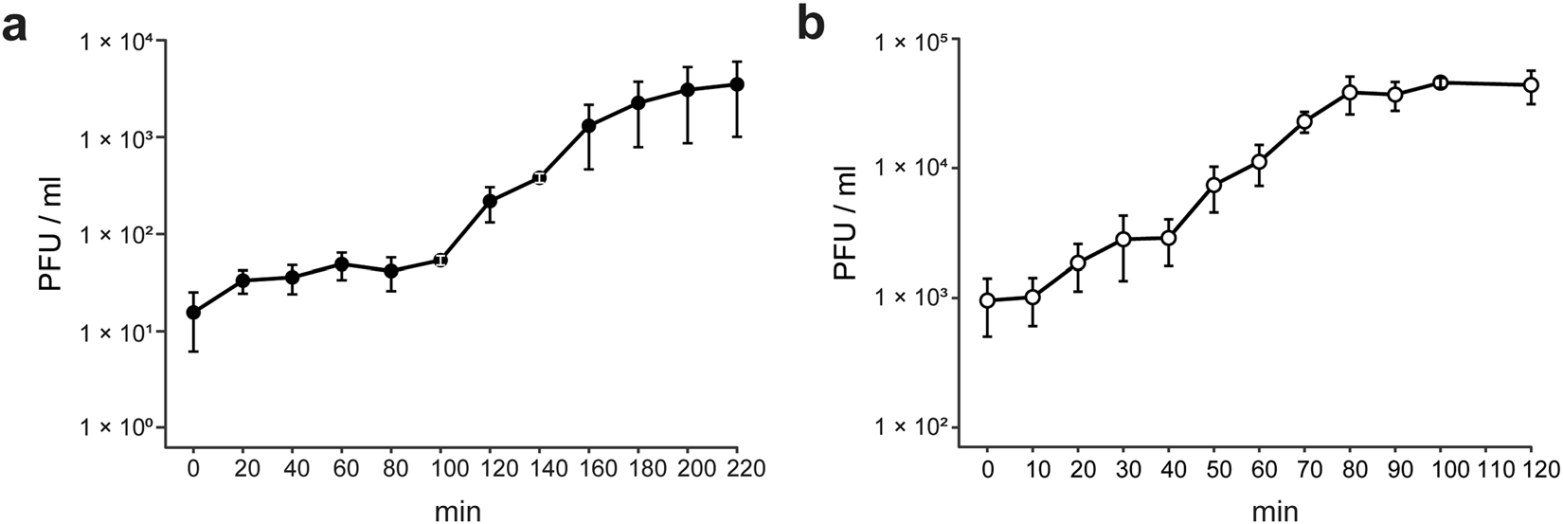
One-step growth curves of phages P26059A (**a**) and P26059B (**b**). The one-step growth curves of P26059A and P26059B are shown with error bars indicating the standard error (*n* = 3).

Two phages, P26059A and P26059B, infecting the *Comamonadaceae* strain IMCC26059 were separately isolated from Lake Soyang. Phage P26059A was isolated in May 2015 using an enrichment culture method, and phage P26059B was isolated in April 2016 through the concentration of phage particles in the lake water followed by screening. Both phages formed plaques on the bacterial lawn plate, indicating active lytic life cycles of both phages. The plaque size for P26059A was approximately 1 mm in diameter and that of P26059B was 5 mm in diameter. When their morphology was observed under transmission electron microscopy (TEM), the two phages were revealed to have different morphologies as well. P26059A belonged to the family *Siphoviridae* with a long tail of 153 nm in length and 62 nm head in diameter (Fig. 1a). Meanwhile, P26059B appeared to be a member of the family *Podoviridae* with a short tail (9 nm) and an icosahedral shaped head (59 nm in diameter) (Fig. 1b). One-step growth curves for both phages were constructed. The latent periods for P26059A and P26059B were 100 and 10 min each, and their burst sizes were ~70 and ~95, respectively (Fig. 2, Supplementary Fig. S2). Two phages not only showed difference in burst sizes but also in lytic cycle period. While phage P26059B completed its lytic cycle within 110 min, phage P26059A took approximately 220 min. The morphology and one-step growth curves of the two phages indicated that the two phages had distinct phenotypic characteristics, although they shared the same bacterial host.

To determine the host range of the two phages, bacterial strains of phylogenetically-related genera to strain IMCC26059 were infected by phages P26059A and P26059B. Of 12 strains belonging to the genera *Rhodoferax*, *Curvibacter*, *Limnohabitans*, and *Polaromonas* that were tested, none of the bacterial strains except for its original host (IMCC26059) showed infectivity, indicating that phages P26059A and P26059B had a very narrow host range.

### Genomic characteristics of P26059A and P26059B

The genomes of the two phages were sequenced using the Illumina MiSeq platform. The genome size of P26069A was revealed to be 84,008 bp with 43.6% G + C content, and that of P26059B was shown to be 41,344 bp long with 54.3% G + C content. The two phage genomes had little sequence similarity at the nucleotide level. The RAST server predicted 124 protein-coding genes within the P26059A genome, and 46 of them within the P26059B genome (Table 1). For P26059A, out of the 124 predicted protein-coding genes, 72 of them had a significant match in either one of the following databases: NCBI nr, NCBI env-nr, UniProt database, or Conserved Domain Database (CDD). Despite the extensive search, 52 of the genes remained as unknown with its function, leaving them as unique proteins of P26059A (Supplementary Table S1). When P26059B protein coding genes were analyzed in the same way, 31 out of 46 genes had a significant match. The remaining 15 genes were searched upon protein domain databases, but no conserved domains were found. Therefore, known functions could not be assigned to those 15 genes (Supplementary Table S2).

**Table 1:** Genome information of phages P26059A and P26059B.

	P26059A	P26059B
Sequencing library	Paired-end TruSeq library	Paired-end TruSeq library
Sequencing platform	Illumina MiSeq	Illumina MiSeq
Fold coverage	1,205×	4,247×
Genome length	84,008 bp	41,344 bp
G+C%	43.60%	54.30%
No. of coding sequences	124	46
tRNA	2	0
Assembler	SPAdes-3.5.0	SPAdes-3.8.2
Gene calling	RAST ver. 2.0	RAST ver. 2.0
GenBank ID	KY981271	KY981272

**Figure 3:**
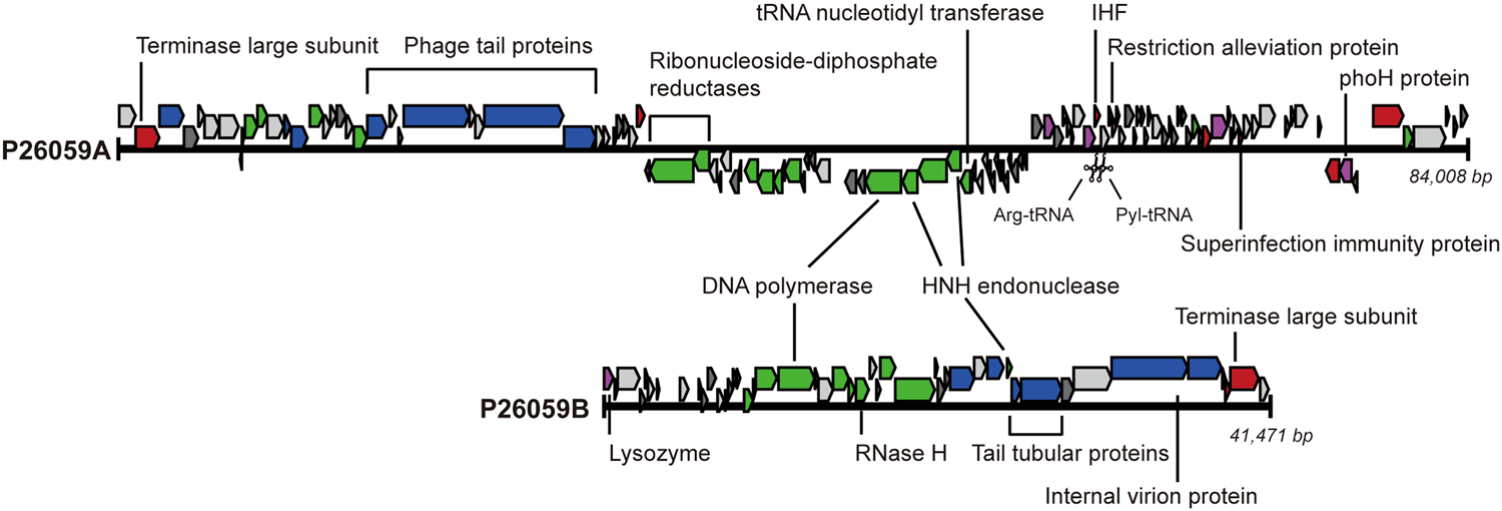
Genome map of P26059A and P26059B. The blue color represents structural genes; the green represents genes related to DNA replication, recombination, and modification; the red represents those related to cell lysis and packaging; the purple color represents auxiliary metabolic genes; and the grey color represents hypothetical genes. The tRNAs are marked with cross signs.

Although the two genomes were not similar to each other at the nucleotide level, they shared a number of genes with similar functions. Both phages carried genes that coded for general bacteriophage proteins such as terminase, capsid, tail proteins, and endonucleases (Fig. 3). One of the most commonly found proteins in double-stranded DNA (dsDNA) bacteriophage is the terminase protein that participates in packaging of the bacteriophage genome into a capsid. Both P26059A and P26059B genomes coded for terminase (ORF2 in both genomes), however, with different features. The terminase large subunit (TerL) of P26059A belonged to the terminase 6 superfamily, while the TerL sequence of P26059B did not belong to a known terminase superfamily but showed high similarity with that of the *Caulobacter* phage Percy. Genes coding for HNH endonucleases, which are also widely distributed bacteriophage proteins, were found in both phage genomes. ORFs 15, 56, and 58 of P26059A and ORF 11 of P26059B were all identified to belong to the HNH endonuclease 3 protein family (PF13392); however, the sequences were too divergent such that all four protein-coding genes were dissimilar from each other. As related to the DNA replication mechanism genes, two phages carried DNA polymerases of different types. P26059A carried a family B DNA polymerase (ORF 55), while P26059B carried a family A DNA polymerase (ORF 27). In addition, P26059B had a DNA-dependent RNA polymerase gene (ORF 18).

**Figure 4:**
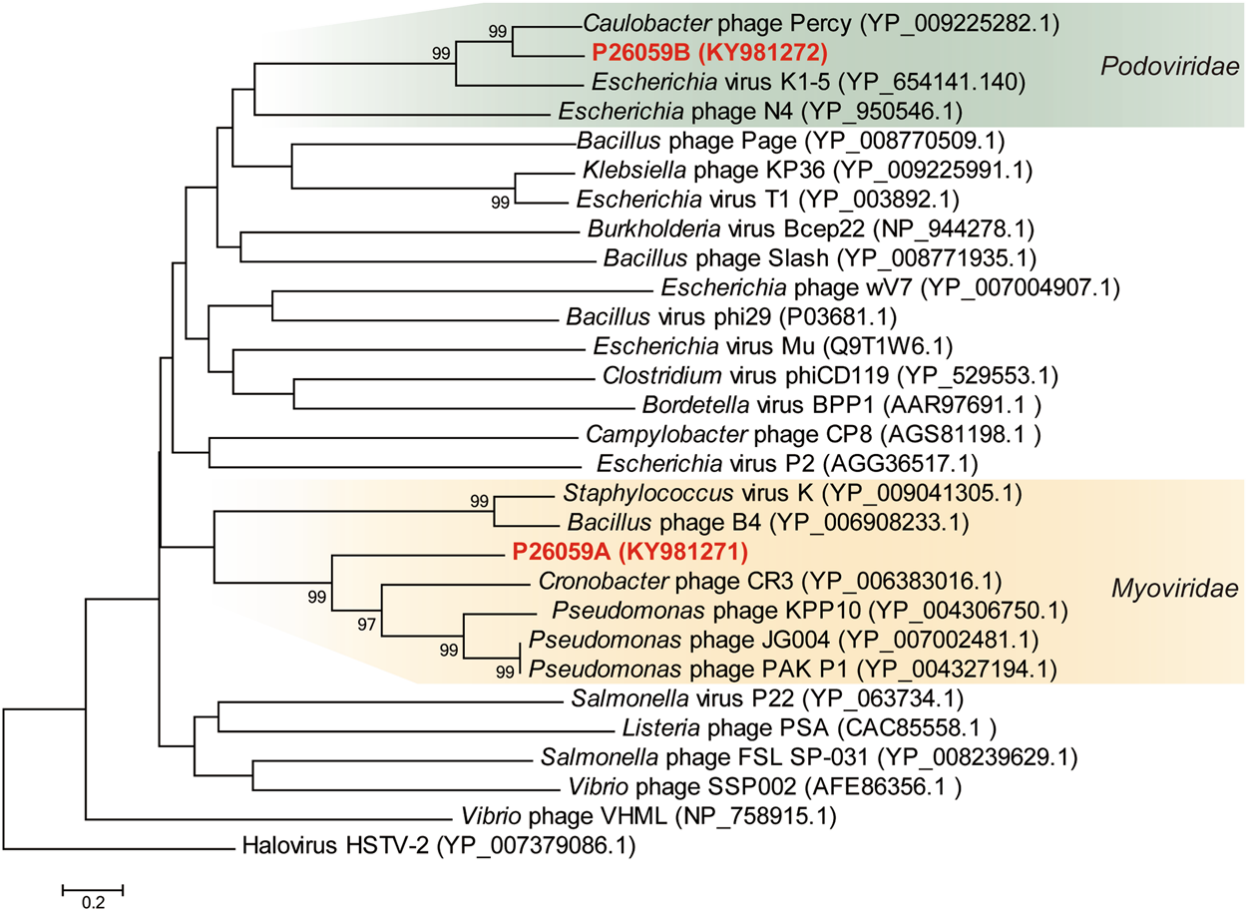
Neighbor-joining phylogenetic tree of the terminase large subunit (TerL) amino acid sequences showing the phylogenetic position of phages P26059A and P26059B. The reference sequences were collected from the NCBI nr database. The tree was constructed using MEGA 6 after performing a sequence alignment using CLUSTAL X. Bootstrap values representing over 70%, calculated based on 1,000 resamplings, are shown at the nodes.

To analyze the phylogenetic relationship between the two phages and other phages deposited in public databases, a phylogenetic tree was constructed using TerL sequences that were carried by both phages. The TerL sequence of the two phages were too divergent from each other to be classified into a monophyletic group (Fig. 4). Phage P26059B was clustered with representative bacteriophages of the family *Podoviridae*. The P26059A TerL protein was clustered with representative TerL proteins of the family *Myoviridae*, although P26059A was morphologically classified as a member of the family *Siphoviridae*. Bacteriophage genomes are often comprised of genome fragments that show similarities to different phages and bacteria, which might have been accumulated from horizontal transfers during infections^29^. The genome of P26059A may also have acquired the *terL* gene through horizontal transfer from other phages or its host.

### Detailed analysis of the P26059A genome

In contrast to the P26059B genome that had typical phage proteins or hypothetical proteins, the P26059A genome harbored more diverse genes. Phage P26059A carried 17 genes related to DNA modification and replication. Among those, 6 endonuclease genes were found, and 4 of them contained a GIY-YIG domain that is widely found in prokaryotes and eukaryotes (ORFs 11, 15, 20, and 58). The GIY-YIG domains are found in endonucleases that function as repair systems of damaged DNA in prokaryotes. Within the bacteriophage genome, it is known to function in cleaving the host DNA to utilize the host nucleotides in phage genome replication^30^, leading to the active replication of phage genomes. The phage P26059A genome also encoded for the PhoH family protein (ORF 117), which is known to be well distributed among marine bacteriophages and cyanophages, and has been used as a phylogenetic marker for phage studies^4,31^. In the phylogenetic analysis using the *phoH* gene, phage P26059A was distantly related with the freshwater *Microcystis* phage Ma-Lmm01 (Supplementary Fig. S3). The *phoH* gene expression within bacteria is known to be induced under phosphate starvation, which would promote the uptake of phosphorous from the environment. Thereby, the *phoH* gene carried by the phage genome is suggested to be expressed in the host cells to promote phosphorous uptake, thus leading to an increase in phosphorus level within the cell to be utilized for phage genome replication. In oligotrophic freshwater environments where phosphorous is typically limiting, such strategy would provide an advantage of faster phage DNA replication with a sufficient amount of resources.

P26059A also carried diverse proteases with different functions. A number of conventional proteases found in phage genomes were also found in the genome of P26059A, such as the serine protease XkdF (ORF 10), peptidase M15 (ORF 35), and cell wall hydrolase (ORF 103). ORF 116 encoded for a caseinolytic protease (ClpP) which is commonly found in bacteria. Within many bacterial cells, ClpP forms a chaperone-protease pair with the ClpA chaperone and takes part in protein degradation. ClpP has been found in bacteriophage genomes as well and it was revealed earlier that this protease acts as a prohead protease for packaging^32^. An integration host factor (IHF) encoded by ORF 82 may also be involved in the packaging of the phage genome into the capsid through the condensation of genomic DNA^33^. Phage P26059A also has genes for defense mechanism. The ORF 85 encoded for a Lar family protein, a restriction alleviation protein^34^, which protects the phage genome from host restriction endonucleases. ORF 108 encoded for a superinfection immunity protein to prevent infections of secondary phages upon infection of the first^35,36^. The presence of such defense mechanisms may provide advantages to P26059A over other phages infecting the identical host.

Within the genome of P26059A, ORF 59 was annotated as tRNA nucleotidyl transferase/poly (A) polymerase, which contributes in tRNA elongation (Supplementary Table S1), hinting for the presence of tRNA genes in the phage genome. Therefore, tRNA was searched using the tRNAscan-SE 2.0 and ARAGORN, and two tRNAs were found. Arg-tRNA was predicted between 61,021 and 61,096 bp by both programs. Meanwhile, Pyl-tRNA, which encodes a tRNA for the non-canonical amino acid pyrrolysine and has been found mostly within archaea and bacteria^37,38^, was also predicted between 61,298 and 61,396 bp.

### Environmental distribution of two phages analyzed using viral metagenomes

**Figure 5:**
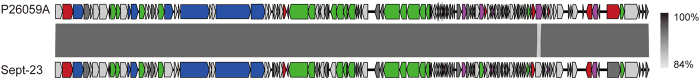
Comparison of the genome of P26059A and its syntenic contig retrieved from the Lake Soyang virome, SY-′15 Sept. Similar regions of 450 bp or longer in length were searched by tBLASTn and displayed by rectangles. Color saturation indicates the degree of amino acid similarity according to color bar at the right.

In order to examine the abundance and distribution of phage populations related to P26059A and P26059B in the environment, viral metagenome samples were used for competitive binning analysis. From the surface layer of Lake Soyang, the original habitat of phages P26059A and P26059B, 6 seasonal virome samples were collected and sequenced using the Illumina MiSeq platform as previously described^17^. The sequences of the Lake Soyang virome were binned against a custom-made database (*see* Methods) using the DIAMOND algorithm^39^, resulting in each of the virome sequences to be assigned to the best-matching protein in the database. Among all of the assigned reads, about 12.5% (SY-′15 Jan.) to 29.4% (SY-′15 Sept.) of the sequences were assigned to viral proteins (Table 2). Proteins of P26059A occupied 1.01% of the viral reads in the SY-′15 Sept. virome sample, ranking the 15^th^ of the most abundant viruses within the sample (Supplementary Table S3). The proportion of P26059A-assigned reads were less than 1% for all other 5 Lake Soyang viromes, and P26059B occupied a much less proportion than P26059A in all samples (Table 2). Within Lake Soyang, phage populations related to either P26059A or P20659B seemed to be present at low frequencies compared to other viruses (Supplementary Tables S3–S9). Especially, P26059B-assigned reads showed a low abundance across all of the virome samples analyzed, indicating that the P26059B phage represented one of the minor bacteriophage populations found in Lake Soyang (Table 2). It is noteworthy that most of the highly-ranked phages in the freshwater viromes (Supplementary Tables S3–S9) were marine-borne phages such as many *Synechococcus*, *Prochlorococcus*, *Pelagibacter*, and *Puniceispirillum* phages, thus more representative freshwater phages infecting frequently occurring freshwater bacteria are needed to be isolated to better understand freshwater microbial ecology comprehensively. Since the proportion of P26059A-assigned reads was the highest in SY-′15 Sept., contigs syntenic to the P26059A genome were searched upon the contigs assembled from the SY-′15 Sept. virome data using tBLASTx. As a result, one contig, Sept-23, appeared to have a remarkable similarity to the P26059A genome, with 99.8% nucleotide sequence similarity (Fig. 5), indicating the existence of the phage group closely related to P26059A.

**Table 2:** Competitive binning of Lake Soyang and Lake Michigan viromes to a reference genome database including P26059A and P26059B.

Virome samples	% of virus/total binned^a^	% of P26059A^b^		% of P26059B^b^	
		%	Rank	%	Rank
SY^c^-′14 Oct.	13.99	0.76	27	0.01	1458
SY-′15 Jan.	12.45	0.38	54	0.01	776
SY-′15 Sept.	29.39	1.01	15	0.01	673
SY-′15 Nov.	24.65	0.18	104	0.01	505
SY-‘16 Feb.	15.97	0.34	55	0.01	691
SY-‘16 May	16.04	0.72	26	0.20	98
LM^d^-SRR1974488	0.40	0.97	15	0.30	67
LM-SRR1974491	2.27	0.44	52	0.09	190
LM-SRR1974494	2.91	2.20	6	0.11	160
LM-SRR1974497	1.95	0.77	21	1.55	7
LM-SRR1974501	1.85	0.64	30	1.21	13
LM-SRR1974503	3.52	0.54	34	0.11	162
LM-SRR1974511	2.15	1.01	16	0.05	281
LM-SRR1974512	1.51	0.79	23	0.17	134
LM-SRR1974513	1.62	1.27	8	0.18	126

^a^Proportion of virus-binned reads among all the binned reads.

^b^Proportion of reads assigned to P26059A or P26059B among all the reads that were binned to viruses.

^c^SY is an abbreviation for Lake Soyang.

^d^LM is an abbreviation for Lake Michigan.

To explore the distribution of P26059A- or P26059B-related phage groups in other freshwater habitats, 9 randomly selected virome data of Lake Michigan were collected and analyzed using the same method. Compared to the Lake Soyang virome, a lesser portion of the binned reads were assigned to viral proteins; 0.4% (SRR197488) to 2.9% (SRR1974494), while most of the reads were assigned to bacterial proteins (Table 2). For three of the virome samples (SRR1974494, SRR1974513, and SRR1974511), P26059A showed a relatively high abundance, ranking 6^th^, 8^th^, and 16^th^ among viruses and contributing 2.2%, 1.3%, and 1.0% of virus-assigned reads, respectively (Table 2, Supplementary Tables S4–S9). Unlike in Lake Soyang, the proportion of P26059B-assigned reads was higher than that of P26059A in two samples, SRR1974497 and SRR1974501, ranking 7^th^ (1.55%) and 13^th^ (1.21%), respectively, which suggested that the distribution patterns of the two phage groups were distinctively different from Lake Soyang.

## Conclusion

In this study, we have successfully isolated two novel lytic phages that infected a single host bacterial strain of the family *Comamonadaceae* from Lake Soyang, an oligotrophic lake located in South Korea, and obtained their whole genome sequences. The two phages showed clear morphological difference, therefore were classified into two different families of the order *Caudovirales*: *Siphoviridae* (P26059A) and *Podoviridae* (P26059B). The genome sequences of the two phages were also very different in length (84.0 kb and 41.5 kb for P26059A and P26059B, respectively), and showed little sequence similarity, although a number of typical phage proteins, such as terminase, were predicted in both genomes. The P26059A genome carried auxiliary metabolic genes including *phoH* and *clpP* that may promote phage amplification. Albeit at low proportions, sequences similar to the two phage genomes were consistently detected in the virome data analyzed in this study. Especially P26059A, which appeared to be more abundant than P26059B, had a seasonal preference for the summer (SY-′15 Sept.). Moreover, a contig highly similar and syntenic to the P26059A genome was also found from the SY-′15 Sept. virome, implying the presence of a phage population sharing much of their genomic contents with P26059A. Prevalence of phages P26059A and P26059B in Lake Soyang indicated a consistent lytic infection of *Comamonadaceae* strain IMCC26059 by the two phages, which might control the population size of this bacterial group. The finding that many characteristics including morphology, genome features, and ecological abundance were different between the two phages sharing the identical host implies that physical and genomic information on phages themselves was not sufficient to infer putative hosts. Thus, along with numerous viral metagenome studies, the isolation and identification of phages infecting diverse bacterial strains must be performed for better interpretation and classification of novel phage genomes retrieved from immense virome data. This study represents one of the very few reports on freshwater phages infecting the family *Comamonadaceae*, an abundant and ubiquitous freshwater bacterial group. Therefore, the two phages, P26059A and P26059B, are expected to contribute to future research on freshwater phages.

## Methods

### Isolation of bacteriophages P26059A and P26059B from Lake Soyang

In April 2014, a *Comamonadaceae* strain named IMCC26059 was isolated from Lake Soyang, which is located in South Korea (37°56′50.4″ N, 127°49′08.4″ E). Strain IMCC26059 was grown and maintained on R2A agar (Becton, Dickenson and Company, Franklin Lakes, NJ, USA) at 20 °C and used as a host to screen for its bacteriophages. In May 2015, surface water from Lake Soyang was collected and brought to the lab at 4 °C. The water sample was filtered through a 0.2-μm PES membrane filter (Merck Millipore, Darmstadt, Germany)^7^ to remove large particles and bacteria, and retain only particles smaller than 0.2-μm in diameter, which was mostly comprised of viral particles. To 400 ml of filtered water sample, 100 ml of 5 × R2A broth (MB Cell, Los Angeles, CA, USA) and liquid culture of IMCC26059 were added to enrich probable bacteriophages infecting IMCC26059 as described previously^26^. From this enrichment culture, a plaque-forming lytic phage was successfully isolated and it was named as P26509A. In June 2016, another surface water sample was collected from the identical site. After filtering the water sample through a 0.2-μm PES membrane filter, 1 L of the water sample was concentrated to approximately 12 ml by ultrafiltration using a 30 kDa Centrifugal Device (Pall Corporation, New York, USA). The sample was filtered through a 0.2-μm Acrodisc® Syringe Filter for sterilization. Ten microliters of the sample were spotted on a IMCC26059 bacterial lawn plate and plaques were obtained from the spotted regions. The plaque was retrieved and purified through a series of double agar layer (DAL) plating and named as P26059B.

### Determination of one-step growth curves and host range of P26059A and P26059B

One-step growth curves for P26059A and P26059B were generated using exponentially growing IMCC26059 liquid culture and phage stocks (~1 × 10^8^ cells/ml for IMCC26059 and 2.43 × 10^8^ PFU/ml and 7.98 × 10^8^ PFU/ml for P26059A and P26059B, respectively) prepared in SM buffer (50 mM Tris-HCl, pH 7.5; 100 mM NaCl; 10 mM MgSO_4_·7H_2_O; 0.01% gelatin). The phage stocks were each inoculated to 10 ml of host liquid culture at a multiplicity of infection (MOI) of 0.05. The mixtures were incubated in a shaking incubator at 20 °C and 100 rpm for 10 min. After incubation, cells infected with phage particles were washed twice by centrifuging at 2,000 × g for 20 min at 4 °C and resuspending in 1 ml of SM buffer, to remove unabsorbed phage particles. The incubated samples were diluted by 10^−4^-fold and were placed in a shaking incubator for 3 hours and 40 min. During the incubation, the liquid culture was withdrawn every 20 min (10 min for P26059B) and was used for a plaque assay based on the DAL method in triplicate. The plaques were counted after overnight incubation at 20 °C, and the obtained plaque numbers were used to construct one-step growth curves. Burst sizes of the two phages were estimated by calculating the ratio of maximum titer of the phage to the infection centers (number of plaques at latent period)^40^.

Host range for phages P26059A and P26059B was determined by observing plaque formation after dropping 10 μl of phage stock onto fresh bacterial lawn plates of phylogenetically-related bacterial strains to the original host, IMCC26059 (Supplementary Fig. S1). The bacterial strains used are as follows*: Rhodoferax saidenbachensis* (DSM 22694), *R. fermentans* (KACC 15304), *Curvibacter fontanous* (KCTC 42832), *C. lanceolatus* (KACC 11655), *C. delicatus* (KACC 12205), *C. gracilis* (KACC 11677), *Limnohabitans planktonicus* (DSM 21594), *L. curvus* (DSM 21645), *L. parvus* (DSM 21592), *L. australis* (DSM 21646), *Polaromonas jejuensis* (KCTC 12508), and *P. aquatica* (KCTC 11696).

### Amplification and concentration of phages

For DNA extraction and TEM sample preparation, the phages were amplified and concentrated according to the ‘Molecular Cloning: A Laboratory Manual’^41^ with minor modifications. Phage P26059A lysate was prepared through inoculation of the P26059A stock with a liquid culture of IMCC26059 in 400 ml of R2A broth with a MOI of 0.1. For phage P26059B, 10 confluent DAL plates with propagated phages were prepared. For phage particle extraction from the plaques, 5 ml of SM buffer were added to each plate and the plates were then incubated on a gyratory shaker at 4 °C. After an overnight incubation, the SM buffer was retrieved. Ten milliliters of chloroform was added to approximately 50 ml of SM buffer retrieved and vigorously vortexed for 5 min. Then the sample was centrifuged at 3,000 × *g* for 30 min and only the top layer was collected for further procedures. To both phage lysate samples, 1 μg ml^−1^ of DNase I and RNase A were added and incubated at 30 °C for 30 min, followed by the addition of 0.06 g of NaCl per ml. To flocculate phage particles, polyethylene glycol (PEG) 8000 (Sigma-Aldrich, St. Louis, MO, USA) was added to a final concentration of 10% (w/v) and incubated in 4 °C for overnight. After incubation, the samples were centrifuged at 11,000 × *g* for 40 min to pellet the phage particles. The phage pellets were resuspended in 3–5 ml of SM buffer, and an equal volume of chloroform was treated to remove PEG. The samples were further concentrated by ultracentrifugation at 45,000 × *g* for 2 hrs (Beckman Coulter L-90K ultracentrifuge with a SW 50 Ti rotor) and the obtained phage pellets were resuspended in 100 μl of SM buffer for further procedures.

### Morphological analysis of two phages using TEM

The phage concentrates from above were adsorbed onto formvar and carbon-coated copper grids. The grids were negatively stained using 2% uranyl acetate^42^ and were observed under a transmission electron microscope (CM200; Phillips, Amsterdam, Netherlands). After observation, taxonomic classification of phages were made based on their morphology^43^.

### Whole genome sequencing and phylogenetic analyses

Genomic DNA was extracted from the phage concentrates using the Qiagen DNeasy Blood and Tissue Kit (Qiagen, Hilden, Germany) according to the manufacturer’s instructions. Whole genome sequencing for the phage genomes was performed by ChunLab, Inc. (Seoul, South Korea). The sequencing libraries were constructed using the TruSeq DNA sample preparation kit and were sequenced using an Illumina MiSeq system with 2 × 300-bp paired-end reads. Raw sequencing reads for P26059A and P26059B were assembled using SPAdes versions 3.5.0 and 3.8.2, respectively^44^. For both phages, the complete genome sequences were obtained as single circular contigs and were annotated by the RAST server^45^. Each predicted gene was analyzed by BLASTP against NCBI’s nr and env-nr protein databases^46^ for their function prediction, and only the results with e-values less than 0.001 were accepted. The protein coding genes that did not have a predicted function were further analyzed using the UniProt database^47^, Pfam database^48^, and Conserved Domain Database (CDD)^49^. Then their functions were predicted based on the protein domain found with an e-value threshold of 0.001. The tRNAs were searched using tRNAscan-SE v. 2.0^50^ and ARAGORN v. 1.2.38^51^, which were available online.

Based on the amino acid sequences obtained from above, phylogenetic analyses for the two phages were made. To construct the phylogenetic tree, sequences for the terminase large subunit (TerL) were selected, which is the most widely found and universally used genetic marker for the order *Caudovirales*. The terminase sequences of the two phages were aligned with those of other reference bacteriophages within the order *Caudovirales*, which were collected from the NCBI nr database, using CLUSTALX^52^ implanted in the MEGA 6^53^. A neighbor-joining phylogenetic tree for terminase large subunit amino acid sequences was constructed using the Kimura-2 parameter model. The robustness of the tree topology was assessed by bootstrap analyses based on 1,000 random resamplings. The phylogenetic position of the PhoH protein encoded in the P26059A genome was also inferred by the same method used for TerL phylogeny.

### Comparative analysis of the P26059A and P26059B genomes and freshwater viromes

In order to analyze the distribution and abundance of phage populations related to the two phages, a competitive binning analysis was performed for 6 viromes of Lake Soyang (PRJEB15535)^17^, the original habitat of the phages, and 9 viromes of Lake Michigan (PRJNA248239)^54^ following the protocol of Moon *et al*.^17^. In brief, a search database was constructed by the addition of proteins predicted in the two phages to all viral and bacterial (non-redundant) proteins of the NCBI RefSeq database (release 79). Sequencing reads of the viromes were assigned to the best-matching protein of the search database using the DIAMOND version 0.8.36 (–query-gencode 11 –single-domain)^39^. All of the reads that were assigned to bacteria were disregarded and only those assigned to bacteriophage and viruses were considered for analysis. To search for viral metagenomic contig syntenic to the P26059A genome, virome data from Lake Soyang (’15 Sept.) were assembled using SPAdes version 3.8.2. The similarities between the assembled contigs (≥10 kb) and the P26059A genome were estimated based on total bitscores from tBLASTx analysis, and contigs showing higher bitscores were selected. The overall synteny between the selected contigs and the P26059A genome was demonstrated using EasyFig^55^ based on the tBLASTn comparison.

### Genome sequence accession numbers

The genome sequences for phages P26059A and P26059B were deposited in the NCBI GenBank database with accession numbers of KY981271 and KY981272, respectively.

## Electronic supplementary material

Supplementary information
