# Supplementary material for: Genomic and ecological study of two distinctive freshwater bacteriophages infecting a Comamonadaceae bacterium
Source: Sci Rep. 2018 May 22;8:7989. doi: 10.1038/s41598-018-26363-y (PMC5964084; doi:10.1038/s41598-018-26363-y)
Supplement: Supplementary file 1 — Supplementary information [file 41598_2018_26363_MOESM1_ESM.docx]

**Supplementary Information**

Genomic and ecological study of two distinctive freshwater bacteriophages infecting a *Comamonadaceae* bacterium

Kira Moon, Ilnam Kang, Suhyun Kim, Sang-Jong Kim, and Jang-Cheon Cho

**Supplementary Fig. S1.** Neighbor-joining phylogenetic tree of 16S rRNA gene sequences showing the phylogenetic position of the host strain IMCC26059 within the family *Comamonadaceae*

**Supplementary Fig. S2.** One-step growth curves of phages P26059A and P26059B

**Supplementary Fig. S3.** Neighbor-joining phylogenetic tree of PhoH protein sequences. Phage P26059A is indicated in red. The reference sequences were collected from Pfam database

**Supplementary Table S1.** Annotation of proteins predicted in the P26059A genome

**Supplementary Table S2.** Annotation of proteins predicted in the P26059B genome

**Supplementary Table S3.** List of the most highly-assigned viruses in the competitive binning analysis of SY-'15 Sept. virome collected from Lake Soyang

**Supplementary Table S4.** List of the most highly-assigned viruses in the competitive binning analysis of Lake Michigan virome (SRR1974488)

**Supplementary Table S5.** List of the most highly-assigned viruses in the competitive binning analysis of Lake Michigan virome (SRR1974494)

**Supplementary Table S6.** List of the most highly-assigned viruses in the competitive binning analysis of Lake Michigan virome (SRR1974497)

**Supplementary Table S7.** List of the most highly-assigned viruses in the competitive binning analysis of Lake Michigan virome (SRR1974501)

**Supplementary Table S8.** List of the most highly-assigned viruses in the competitive binning analysis of Lake Michigan virome (SRR1974511)

**Supplementary Table S9.** List of the most highly-assigned viruses in the competitive binning analysis of Lake Michigan virome (SRR1974513)


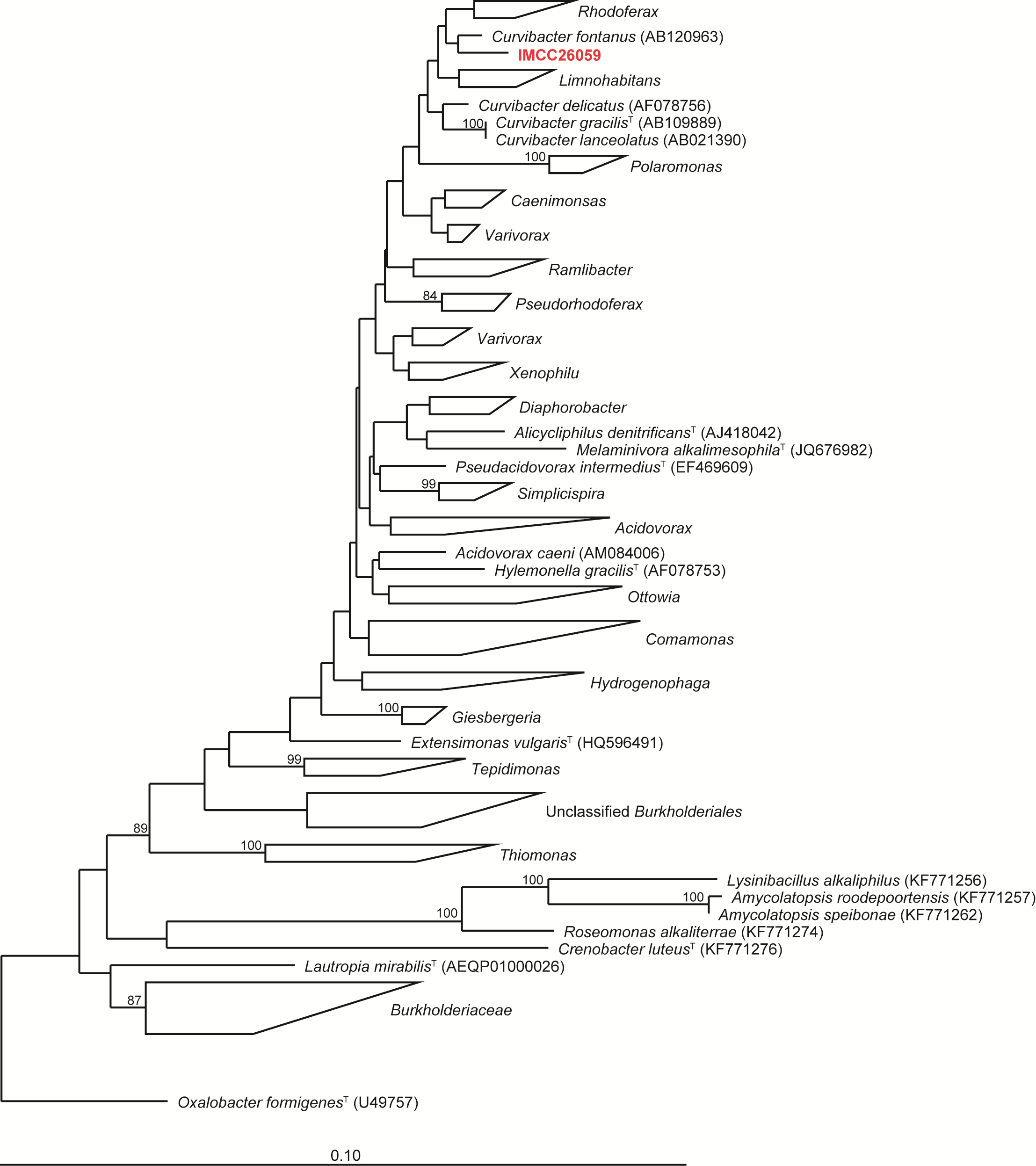


**Supplementary Fig. S1.** Neighbor-joining phylogenetic tree of 16S rRNA gene sequences showing the phylogenetic position of the host strain IMCC26059 within the family *Comamonadaceae*. The tree was constructed with the Jukes-Cantor distance based on the alignment of SILVA ribosomal RNA gene database using the ARB program. Bootstrap values representing over 70%, calculated based on 1,000 resamplings, are shown at the nodes.


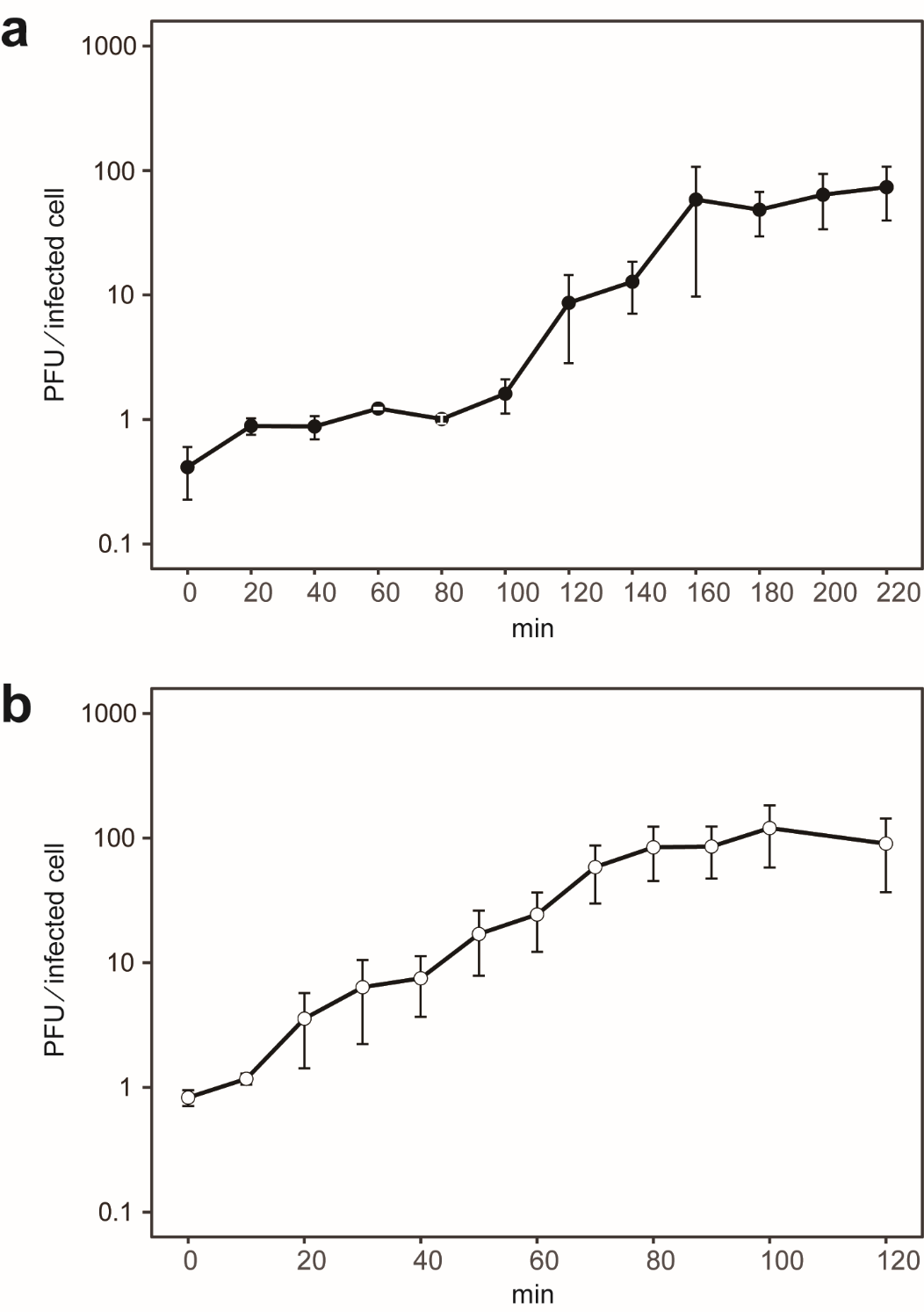


**Supplementary Fig. S2.** One-step growth curves of phages P26059A (a) and P26059B (b). Number of infection centers for phage P26059A was estimated by averaging plaque numbers obtained at time points 20, 40, 60, and 80 and that of phage P26059B was obtained by taking average of plaque numbers obtained at times 0 and 10.


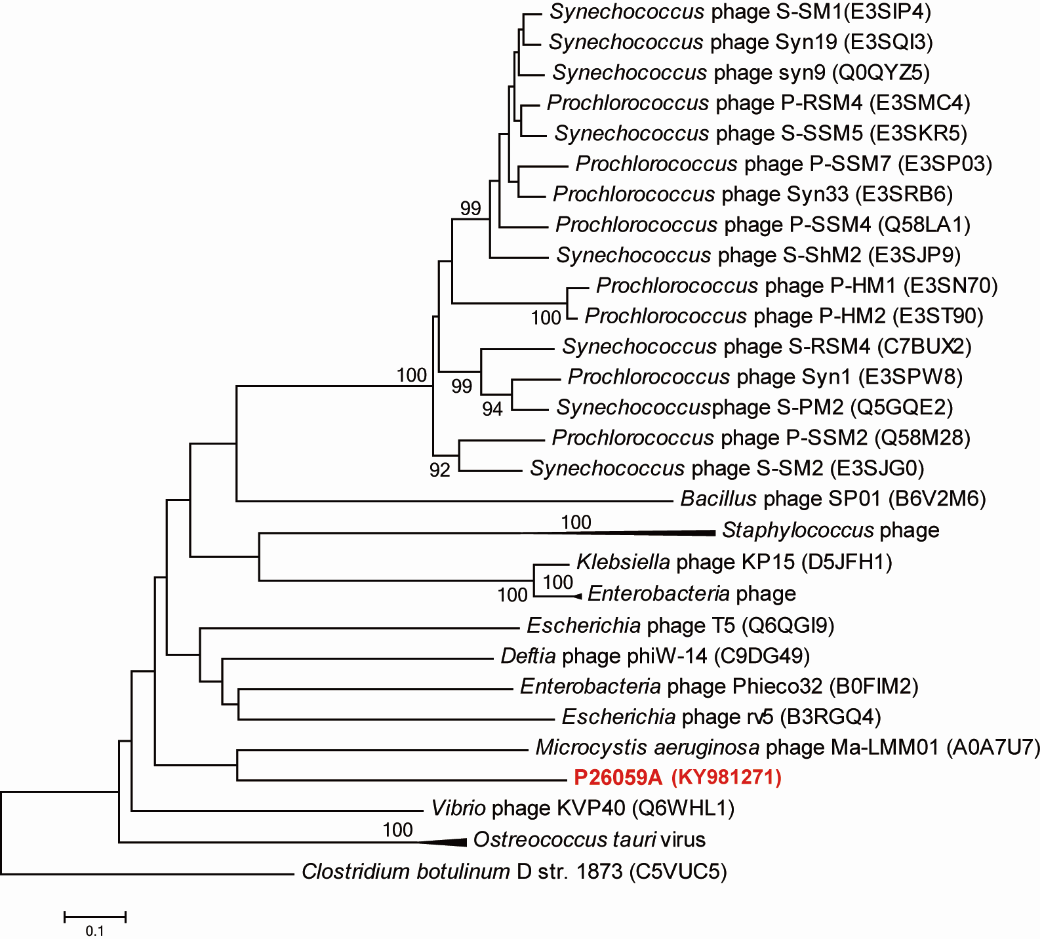


**Supplementary Fig. S3.** Neighbor-joining phylogenetic tree of PhoH protein sequences. Phage P26059A is indicated in red. The reference sequences were collected from Pfam database. The tree was constructed using MEGA 6 after performing sequence alignment using CLUSTAL X. Bootstrap values representing over 70%, calculated based on 1,000 resamplings, are shown at the nodes.

**Supplementary Table S1.** Annotation of proteins predicted in the P26059A genome

| **ORF** | **Position** | **Strand** | **Predicted function (Best BLAST match)** | **E-value** | **% Ident.** | **% Cover** | **DB (Accession No.)** |
| --- | --- | --- | --- | --- | --- | --- | --- |
| 1 | 51-1067 | + | Hypothetical protein (*Vibrio owensii*) | 3.00E-26 | 27% | 95% | nr (WP_039839651.1) |
| 2 | 1078-2550 | + | Terminase large subunit (*Pseudomonas* phage JG004) | 1.00E-101 | 38% | 96% | nr (YP_007002481.1) |
| 3 | 2568-4064 | + | Structural protein (*Pseudomonas* phage KPP10) | 5.00E-119 | 40% | 97% | nr (YP_004849306.1) |
| 4 | 4084-4959 | + | No significant similarity found |  |  |  |  |
| 5 | 4980-5345 | + | Hypothetical protein (*Sinorhizobium medicae*) | 3.00E-20 | 52% | 99% | nr (WP_011975491.1) |
| 6 | 5369-6277 | + | Hypothetical protein (*Rhodobacter* sp. SW2) | 3.00E-12 | 30% | 96% | nr (EEW23746.1) |
| 7 | 6296-7480 | + | Hypothetical protein (*Brevundimonas* sp. Root1279) | 2.00E-26 | 30% |  | Swiss-prot (A0A0Q7E659) |
| 8 | 7695-7576 | - | No significant similarity found |  |  |  |  |
| 9 | 7684-7806 | + | No significant similarity found |  |  |  |  |
| 10 | 7886-8653 | + | Putative phage serine protease XkdF | 1.31E-27 |  |  | CDD (cl24270) |
| 11 | 8661-9266 | + | Putative endonuclease /GIY-YIG nuclease domain | 2.47E-09 |  |  | CDD (cl15257) |
| 12 | 9263-10288 | + | Hypothetical protein (*Pseudomonas* phage PAK_P1) | 5.00E-38 | 36% | 98% | nr (YP_004327197.1) |
| 13 | 10319-10717 | + | Bacteriophage lambda head decoration protein D (*Rhodobacteraceae* bacterium HLUCCA12) | 1.00E-14 | 39% | 96% | nr (KPQ04613.1) |
| 14 | 10756-11769 | + | Major structural protein (*Pseudomonas* phage KPP10) | 8.00E-83 | 42% | 98% | nr (YP_004306755.1) |
| 15 | 11928-12758 | + | HNH endonuclease family protein (*Pseudomonas* phage phiPto-bp6g) | 1.50E-09 | 39% |  | Swiss-prot (G3KB07) |
| 16 | 12767-13192 | + | Hypothetical protein (*Pseudomonas* phage phiPsa374) | 2.30E-16 | 40% |  | Swiss-prot (W8EIG9\) |
| 17 | 13194-13550 | + | Hypothetical protein (Marine metagenome) | 8.00E-34 | 42% | 98% | env-nr (EBO74624.1) |
| 18 | 13608-14153 | + | No significant similarity found |  |  |  |  |
| 19 | 14165-14569 | + | Hypothetical protein (*Methylovorus glucosotrophus*) | 1.00E-26 | 41% | 98% | nr (WP_015830281.1) |
| 20 | 14659-15465 | + | Putative endonuclease (Uncultured archaeon) | 1.20E-13 | 29% |  | Swiss-prot (D1JFH5\) |
| 21 | 15494-16708 | + | Putative major tail structural protein (*Pseudomonas* phage PaMx74) | 4.00E-44 | 35% | 90% | nr (YP_009199465.1) |
| 22 | 16890-17300 | + | Hypothetical protein (*Burkholderia ubonensis*) | 2.00E-16 | 31% | 97% | nr (WP_059735913.1) |
| 23 | 17434-17661 | + | Hypothetical protein (*Rhizobium tropici*) | 2.00E-06 | 31% | 100% | nr (WP_052227533.1) |
| 24 | 17766-21848 | + | Phage tail tape measure protein lambda (*Pseudomonas syringae* pv. *apii*) | 2.00E-61 | 25% |  | Swiss-prot (A0A0P9J5U) |

**Supplementary Table S1.** Continued

| **ORF** | **Position** | **Strand** | **Predicted function (Best BLAST match)** | **E-value** | **% Ident.** | **% Cover** | **DB (Accession No.)** |
| --- | --- | --- | --- | --- | --- | --- | --- |
| 25 | 21858-22223 | + | Hypothetical protein (*Desulfovibrio vulgaris*) | 2.00E-15 | 39% | 95% | nr (WP_010939433.1) |
| 26 | 22220-22741 | + | Hypothetical protein (*Desulfovibrio vulgaris*) | 2.00E-39 | 46% | 100% | nr (WP_014524470.1) |
| 27 | 22767-27701 | + | Phage-related protein, tail component (*Methyloceanibacter caenitepidi*) | 1.30E-108 | 32% |  | Swiss-prot (A0A0A8K5V0) |
| 28 | 27748-29724 | + | Putative tail fiber protein (*Sinorhizobium* phage phiN3) | 9.00E-37 | 27% |  | Swiss-prot (A0A0R8UEH9) |
| 29 | 29734-30204 | + | Hypothetical protein (*Variovorax paradoxus*) | 5.00E-20 | 46% | 91% | nr (WP_057592238.1) |
| 30 | 30205-30567 | + | Hypothetical protein (*Dechlorosoma suillum*) | 2.40E-13 | 39% |  | Swiss-prot (G8QPT8) |
| 31 | 30820-31011 | + | No significant similarity found |  |  |  |  |
| 32 | 31013-31423 | + | No significant similarity found |  |  |  |  |
| 33 | 31427-31780 | + | No significant similarity found |  |  |  |  |
| 34 | 31798-32289 | + | Hypothetical protein (*Labrenzia alba*) | 9.10E-06 | 29% |  | Swiss-prot (A0A0M6YUJ9) |
| 35 | 32286-32750 | + | Peptidase M15 (*Burkholderia vietnamiensis*) | 2.00E-36 | 48% | 94% | nr (WP_011886401.1) |
| 36 | 33053-32811 | - | Thiol reductase thioredoxin (*Halosimplex carlsbadense*) | 1.00E-10 | 40% | 95% | nr (WP_006884752.1) |
| 37 | 35798-33093 | - | Ribonucleoside-diphosphate reductase (*Acinetobacter* phage YMC13/03/R2096) | 0.00E+00 | 42% | 99% | nr (YP_009146801.1) |
| 38 | 36780-35791 | - | RnR beta subunit (*Shewanella* sp. phage 1/40) | 6.00E-112 | 55% | 94% | nr (YP_009104029.1) |
| 39 | 37253-36792 | - | Hypothetical protein (*Pseudomonas* phage KPP10) | 3.90E-04 |  |  | Swiss-prot (D6RRL7) |
| 40 | 37516-37340 | - | No significant similarity found |  |  |  |  |
| 41 | 38112-37516 | - | Hypothetical protein (Marine sediment metagenome) | 2.00E-14 | 38% | 52% | Swiss-prot (A0A0F9K2S2) |
| 42 | 38605-38105 | - | No significant similarity found |  |  |  |  |
| 43 | 38759-38631 | - | No significant similarity found |  |  |  |  |
| 44 | 39794-39000 | - | Putative DNA-binding protein 2 (*Bacillus* phage B4) | 1.00E-16 | 27% | 85% | nr (YP_006908397.1) |
| 45 | 40783-39779 | - | DNA polymerase I (Uncultured Mediterranean phage uvMED) | 5.00E-32 | 31% | 95% | nr (BAQ92511.1) |
| 46 | 41326-40793 | - | Recombination endonuclease VII (*Enterobacteria* phage vB_KleM-RaK2) | 2.50E-13 | 35% |  | Swiss-prot (H6X3P3) |
| 47 | 41471-41286 | - | No significant similarity found |  |  |  |  |

**Supplementary Table S1.** Continued

| **ORF** | **Position** | **Strand** | **Predicted function (Best BLAST match)** | **E-value** | **% Ident.** | **% Cover** | **DB (Accession No.)** |
| --- | --- | --- | --- | --- | --- | --- | --- |
| 48 | 42458-41481 | - | Putative exodeoxyribonuclease (*Klebsiella* phage vB_KpnM_KB57) | 1.00E-44 | 33% | 94% | nr (YP_009187734.1) |
| 49 | 42718-42506 | - | No significant similarity found |  |  |  |  |
| 50 | 42972-42721 | - | No significant similarity found |  |  |  |  |
| 51 | 43374-43021 | - | No significant similarity found |  |  |  |  |
| 52 | 44267-43443 | - | Gp205 (*Bacillus* phage G) | 1.00E-28 | 36% |  | Swiss-prot (G3MBS1) |
| 53 | 45928-45257 | - | No significant similarity found |  |  |  |  |
| 54 | 46462-46043 | - | No significant similarity found |  |  |  |  |
| 55 | 48724-46520 | - | Putative DNA polymerase (*Vibrio* phage vB_VchM-138) | 2.00E-135 | 37% | 93% | nr (YP_007006391.1) |
| 56 | 49735-48824 | - | HNH endonuclease (*Salmonella enterica* subsp. *enterica* serovar Give str. 564) | 2.00E-25 | 37% | 90% | nr (ESH10154.1) |
| 57 | 51554-49806 | - | Putative DNA primase/helicase (*Pseudomonas* phage PAK_P1) | 2.00E-141 | 42% | 94% | nr (YP_004327242.1) |
| 58 | 52425-51565 | - | HNH homing endonuclease (*Salmonella* phage Shivani) | 2.30E-11 | 39% |  | Swiss-prot (A0A0A7TWE4) |
| 59 | 53035-52436 | - | tRNA nucleotidyl transferase/ poly (A) polymerase (*Escherichia* phage phAPEC8) | 2.00E-10 | 28% | 79% | nr (YP_007348551.1) |
| 60 | 53158-53045 | - | No significant similarity found |  |  |  |  |
| 61 | 53354-53160 | - | No significant similarity found |  |  |  |  |
| 62 | 53794-53456 | - | Hypothetical protein (*Achromobacter xylosoxidans*) | 1.00E-16 | 45% | 91% | nr (AKP88981.1) |
| 63 | 54099-53791 | - | Hypothetical protein (*Colwellia* phage 9A) | 9.10E-06 | 47% |  | Swiss-prot (I3UMK3) |
| 64 | 54287-54096 | - | Hypothetical protein (Marine sediment metagenome) | 3.20E-21 | 66% |  | Swiss-prot (A0A0F8XL42) |
| 65 | 54553-54284 | - | No significant similarity found |  |  |  |  |
| 66 | 54771-54550 | - | Hypothetical protein (*Synechococcus* phage S-EIVl) | 2.00E-09 | 39% |  | Swiss-prot (A0A0C4K633) |
| 67 | 54932-54780 | - | No significant similarity found |  |  |  |  |
| 68 | 55109-54948 | - | Tol-Pal system protein YbgF | 1.74E-05 |  |  | CDD (PRK10803) |
| 69 | 55387-55112 | - | No significant similarity found |  |  |  |  |
| 70 | 55578-55390 | - | No significant similarity found |  |  |  |  |
| 71 | 55997-55581 | - | No significant similarity found |  |  |  |  |

**Supplementary Table S1.** Continued

| **ORF** | **Position** | **Strand** | **Predicted function (Best BLAST match)** | **E-value** | **% Ident.** | **% Cover** | **DB (Accession No.)** |
| --- | --- | --- | --- | --- | --- | --- | --- |
| 72 | 56301-55987 | - | No significant similarity found |  |  |  |  |
| 73 | 56544-56404 | - | No significant similarity found |  |  |  |  |
| 74 | 56891-57502 | + | No significant similarity found |  |  |  |  |
| 75 | 57650-58270 | + | Putative phosphoesterase (*Pseudomonas* phage vB_PaeM_C2-10_Ab1) | 9.00E-40 | 42% | 90% | nr (YP_007236845.1) |
| 76 | 58267-58791 | + | No significant similarity found |  |  |  |  |
| 77 | 58794-59057 | + | No significant similarity found |  |  |  |  |
| 78 | 59054-59311 | + | No significant similarity found |  |  |  |  |
| 79 | 59311-59460 | + | No significant similarity found |  |  |  |  |
| 80 | 59472-60125 | + | Hypothetical protein (Marine sediment metagenome) | 1.80E-10 | 38% |  | Swiss-prot (A0A0F9MA30) |
| 81 | 60118-60783 | + | Thymidylate synthase complementing protein (Uncultured bacterium) | 5.00E-76 | 58% | 90% | nr (AIA11069.1) |
| 82 | 60783-61100 | + | Integration host factor, IHF | 8.03E-05 |  |  | CDD (cd13832) |
| 83 | 61174-61653 | + | Hypothetical protein (*Acidovorax* sp. Root70) | 9.60E-06 | 41% |  | Swiss-prot (A0A0Q8LHN8) |
| 84 | 61650-61856 | + | No significant similarity found |  |  |  |  |
| 85 | 61853-62125 | + | Restriction alleviation protein, Lar family | 8.01E-04 | 33% |  | CDD (cl08047) |
| 86 | 62127-62303 | + | No significant similarity found |  |  |  |  |
| 87 | 62305-62664 | + | Hypothetical protein (Marine sediment metagenome) | 3.10E-07 |  |  | Swiss-prot (A0A0F9K1M2) |
| 88 | 62676-63194 | + | No significant similarity found |  |  |  |  |
| 89 | 63205-63369 | + | No significant similarity found |  |  |  |  |
| 90 | 63378-63671 | + | No significant similarity found |  |  |  |  |
| 91 | 63675-63875 | + | No significant similarity found |  |  |  |  |
| 92 | 63868-64089 | + | Hypothetical protein (*Morganella morganii*) | 9.00E-06 | 41% | 91% | nr (WP_049246392.1) |
| 93 | 64089-64262 | + | No significant similarity found |  |  |  |  |
| 94 | 64394-64981 | + | Putative bacteriophage protein (*Burkholderia pseudomallei*) | 3.50E-55 | 60% |  | Swiss-prot (A0A0H5L1B2) |
| 95 | 64984-65349 | + | No significant similarity found |  |  |  |  |

**Supplementary Table S1.** Continued

| **ORF** | **Position** | **Strand** | **Predicted function (Best BLAST match)** | **E-value** | **% Ident.** | **% Cover** | **DB (Accession No.)** |
| --- | --- | --- | --- | --- | --- | --- | --- |
| 96 | 65399-65563 | + | No significant similarity found |  |  |  |  |
| 97 | 65566-65946 | + | No significant similarity found |  |  |  |  |
| 98 | 65943-66125 | + | No significant similarity found |  |  |  |  |
| 99 | 66198-66455 | + | No significant similarity found |  |  |  |  |
| 100 | 66469-66819 | + | No significant similarity found |  |  |  |  |
| 101 | 66866-67249 | + | Ribosomal protein L7/L12, ClpS-like protein | 6.10E-04 |  |  | Pfam (pfam00542) |
| 102 | 67264-67497 | + | No significant similarity found |  |  |  |  |
| 103 | 67510-68001 | + | Cell wall hydrolase (*Paracoccus sphaerophysae*) | 2.00E-19 | 35% | 93% | nr (WP_036720692.1) |
| 104 | 68072-69037 | + | Predicted acyltransferase (*Chlamydia trachomatis*) | 9.00E-63 | 44% | 95% | nr (CRH65641.1) |
| 105 | 69066-69239 | + | No significant similarity found |  |  |  |  |
| 106 | 69229-69357 | + | No significant similarity found |  |  |  |  |
| 107 | 69419-69751 | + | Hypothetical protein (*Campylobacter* sp. FOBRC14) | 7.00E-05 | 32% | 93% | nr (WP_009650654.1) |
| 108 | 69754-69981 | + | Superinfection immunity protein (*Burkholderia* sp. RPE67) | 9.00E-12 |  |  | Pfam (pfam14373) |
| 109 | 70061-70495 | + | Hypothetical protein (Marine metagenome) | 3.00E-07 | 32% | 99% | env-nr (EBQ27659.1) |
| 110 | 70568-70999 | + | Hypothetical protein (*Hafnia alvei* FB1) | 5.30E-17 | 48% |  | Swiss-prot (A0A097R3K3) |
| 111 | 71073-71990 | + | Phage protein (*Escherichia coli*) | 1.00E-30 | 29% | 93% | nr (WP_021549731.1) |
| 112 | 72647-72907 | + | No significant similarity found |  |  |  |  |
| 113 | 72987-73220 | + | No significant similarity found |  |  |  |  |
| 114 | 73284-73991 | + | Hypothetical protein (*Pseudomonas* phage PAK_P1) | 8.00E-80 | 53% | 94% | nr (YP_004327286.1) |
| 115 | 74699-74860 | + | No significant similarity found |  |  |  |  |
| 116 | 75992-75186 | - | Caseinolytic protease | 7.23E-17 |  |  | CDD (cd07016) |
| 117 | 76763-76002 | - | Putative PhoH family protein (*Escherichia* phage phAPEC8) | 1.00E-37 | 31% | 98% | nr (YP_007348464.1) |
| 118 | 77107-76835 | - | No significant similarity found |  |  |  |  |
| 119 | 78123-79991 | + | Putative membrane-anchored cell surface protein (*Burkholderia glumae* PG1) | 9.10E-05 | 22% |  | Swiss-prot (A0A0B6S8R5) |

**Supplementary Table S1.** Continued

| **ORF** | **Position** | **Strand** | **Predicted function (Best BLAST match)** | **E-value** | **% Ident.** | **% Cover** | **DB (Accession No.)** |
| --- | --- | --- | --- | --- | --- | --- | --- |
| 120 | 80032-80592 | + | HNH endonuclease | 1.93E-05 |  |  | Pfam (pfam13392) |
| 121 | 80644-82602 | + | Hypothetical protein (*Klebsiella pneumoniae* subsp. *pneumoniae* KP5-1) | 1.30E-16 | 29% |  | Swiss-prot (A0A0M5JR58) |
| 122 | 82653-82847 | + | Hypothetical protein (Marine metagenome) | 1.00E-14 | 49% | 95% | env-nr (ECV37839.1) |
| 123 | 83129-83350 | + | No significant similarity found |  |  |  |  |
| 124 | 83580-83963 | + | No significant similarity found |  |  |  |  |

**Supplementary Table S2.** Annotation of proteins predicted in the P26059B genome

| **ORF** | **Position** | **Strand** | **Predicted function (Best BLAST match)** | **E-value** | **% Ident.** | **% Cover** | **DB (Accession No.)** |
| --- | --- | --- | --- | --- | --- | --- | --- |
| 1 | 510-1 | - | Hypothetical protein (*Acinetobacter guillouiae*) | 2.00E-10 | 42% | 62% | nr (BAP37666.1) |
| 2 | 2289-520 | - | Large terminase (*Caulobacter* phage Percy) | 0.00E+00 | 55% | 100% | nr (YP_009225282.1) |
| 3 | 2597-2286 | - | Putative DNA maturase A (*Caulobacter* phage Cd1) | 7.00E-07 | 41% | 80% | nr (ADD21677.1) |
| 4 | 2778-2584 | - | Phage holin T7 family, holin superfamily II (*Paraburkholderia diazotrophica*) | 6.00E-13 | 47% | 93% | nr (SEI42881.1) |
| 5 | 4900-2828 | - | Tail fiber protein (*Caulobacter* phage Cd1) | 6.00E-19 | 45% | 18% | nr (ADD21674.1) |
| 6 | 9673-4940 | - | Internal virion protein (*Caulobacter* phage Cd1) | 9.00E-139 | 35% | 52% | nr (ADD21673.1) |
| 7 | 12030-9682 | - | Hypothetical protein (*Pseudomonas* phage VSW-3) | 1.00E-26 | 24% | 77% | nr (ANH51101.1) |
| 8 | 12758-12033 | - | No significant similarity found |  |  |  |  |
| 9 | 15296-12768 | - | Tail tubular protein B (*Caulobacter* phage Cd1) | 0.00E+00 | 41% | 99% | nr (ADD21670.1) |
| 10 | 15887-15297 | - | Tail tubular protein A (*Xanthomonas* phage f30-Xaj) | 4.00E-35 | 38% | 96% | nr (AMM44688.1) |
| 11 | 16183-15890 | - | HNH endonuclease (*Desulfosporosinus acididurans*) | 9.00E-21 | 47% | 97% | nr (WP_053006391.1) |
| 12 | 17401-16394 | - | Major capsid-like protein (*Ralstonia* phage RSB1) | 3.00E-119 | 53% | 97% | nr (YP_002213721.1) |
| 13 | 18223-17474 | - | Hypothetical protein (*Burkholderia thailandensis* MSMB43) | 4.00E-36 | 41% | 79% | nr (EIP87426.1) |
| 14 | 19725-18220 | - | Head-to-tail joining protein (*Caulobacter* phage Cd1) | 1.00E-154 | 48% | 93% | nr (ADD21666.1) |
| 15 | 20061-19726 | - | No significant similarity found |  |  |  |  |
| 16 | 20495-20061 | - | No significant similarity found |  |  |  |  |
| 17 | 20662-20492 | - | Hypothetical protein (*Burkholderia ubonensis*) | 1.00E-08 | 53% | 87% | nr (WP_060288674.1) |
| 18 | 23135-20685 | - | DNA-dependent RNA polymerase (*Caulobacter* phage Percy) | 0.00E+00 | 49% | 99% | nr (YP_009225265.1) |
| 19 | 24070-23132 | - | Putative ATP-dependent DNA ligase (*Burkholderia* phage Bp-AMP1) | 1.00E-33 | 32% | 99% | nr (CDK30097.1) |
| 20 | 24314-24063 | - | No significant similarity found |  |  |  |  |
| 21 | 24721-24308 | - | Hypothetical protein (*Candidatus* *Accumulibacter* sp. SK-11) | 2.00E-37 | 61% | 78% | nr (EXI76492.1) |
| 22 | 25577-24798 | - | RNase H superfamily protein (*Burkholderia* sp. BDU5) | 3.00E-96 | 56% | 100% | nr (WP_059473379.1) |
| 23 | 26042-25659 | - | DNA endonuclease VII (*Xylella* phage Prado) | 7.00E-43 | 60% | 94% | nr (YP_008859405.1) |
| 24 | 27006-26017 | - | DNA exonuclease (*Burkholderia* phage JG068) | 1.00E-56 | 41% | 89% | nr (YP_008853860.1) |

**Supplementary Table S2.** Continued

| **ORF** | **Position** | **Strand** | **Predicted function (Best BLAST match)** | **E-value** | **% Ident.** | **% Cover** | **DB (Accession No.)** |
| --- | --- | --- | --- | --- | --- | --- | --- |
| 25 | 27914-27006 | - | Hypothetical protein (*Ralstonia* *solanacearum*) | 2.00E-64 | 47% | 84% | nr (WP_042591591.1) |
| 26 | 28137-27919 | - | No significant similarity found |  |  |  |  |
| 27 | 30585-28150 | - | DNA polymerase (*Caulobacter* phage Cd1) | 0.00E+00 | 53% | 100% | nr (ADD21653.1) |
| 28 | 31892-30585 | - | Putative DNA helicase (*Ralstonia* phage RSJ2) | 5.00E-168 | 55% | 99% | nr (YP_009216554.1) |
| 29 | 32071-31886 | - | No significant similarity found |  |  |  |  |
| 30 | 32625-32068 | - | DNA primase (*Burkholderia cepacia*) | 5.00E-46 | 48% | 90% | nr (WP_060050871.1) |
| 31 | 33092-32865 | - | No significant similarity found |  |  |  |  |
| 32 | 33323-33105 | - | No significant similarity found |  |  |  |  |
| 33 | 33634-33320 | - | No significant similarity found |  |  |  |  |
| 34 | 33816-33631 | - | No significant similarity found |  |  |  |  |
| 35 | 34287-33886 | - | Hypothetical protein (*Vibrio vulnificus*) | 1.00E-15 | 54% | 48% | nr (KOR91322.1) |
| 36 | 34889-34392 | - | No significant similarity found |  |  |  |  |
| 37 | 35280-34900 | - | No significant similarity found |  |  |  |  |
| 38 | 35440-35270 | - | No significant similarity found |  |  |  |  |
| 39 | 36622-36095 | - | Hypothetical protein (*Pseudomonas* phage YMC11/06/C171_PPU_BP) | 2.00E-20 | 34% | 82% | nr (YP_009275032.1) |
| 40 | 38041-37913 | - | No significant similarity found |  |  |  |  |
| 41 | 38611-38231 | - | Hypothetical protein (*Bradyrhizobium* sp. Cp5.3) | 5.00E-08 | 38% | 80% | nr (WP_027554547.1) |
| 42 | 38807-38604 | - | Hypothetical protein (*Vibrio nigripulchritudo* SOn1) | 3.00E-04 | 39% | 83% | nr (CCO46700.1) |
| 43 | 39114-38776 | - | No significant similarity found |  |  |  |  |
| 44 | 40499-39141 | - | Hypothetical protein (*Sphingomonas adhaesiva*) | 1.00E-65 | 40% | 80% | nr (WP_066707580.1) |
| 45 | 40693-40496 | - | Hypothetical protein (*Gemmatimonas* sp. SG8_17) | 3.00E-04 | 51% | 66% | nr (KPJ91942.1) |
| 46 | 41267-40773 | - | Putative lysozyme (*Burkholderia* phage Bp-AMP4) | 9.00E-38 | 53% | 73% | nr (CDL65241.1) |

**Supplementary Table S3.** List of the most highly-assigned viruses in the competitive binning analysis of SY-'15 Sept. virome collected from Lake Soyang.

| Rank | Name | %^a^ |
| --- | --- | --- |
| 1 | *Synechococcus* phage S-SKS1 | 6.86 |
| 2 | *Synechococcus* phage S-SM2 | 6.74 |
| 3 | *Prochlorococcus* phage P-SSM2 | 5.24 |
| 4 | *Pelagibacter* phage HTVC008M | 4.30 |
| 5 | *Pelagibacter* phage HTCC010P | 3.98 |
| 6 | *Streptomyces* phage Jay2Jay | 3.85 |
| 7 | *Prochlorococcus* phage P-TIM68 | 2.96 |
| 8 | *Puniceispirillum* phage HMO-2011 | 2.46 |
| 9 | *Synechococcus* phage ACG-2-14f | 2.04 |
| 10 | *Synechococcus* phage S-SSM7 | 1.92 |
| 11 | *Synechococcus* phage S-CBS4 | 1.75 |
| 12 | *Synechococcus* phage S-PM2 | 1.41 |
| 13 | *Cronobacter* phage vB-CsaM-GAP32 | 1.18 |
| 14 | *Rhodothermus* phage RM378 | 1.02 |
| 15 | **P26059A** | 1.01 |
| 16 | *Synechococcus* phage S-CRM01 | 0.96 |
| 17 | Cyanophage S-RIM50 | 0.94 |
| 18 | *Caulobacter* phage Cr30 | 0.91 |
| 19 | Cyanophage S-TIM5 | 0.82 |
| 20 | Cyanophage KBS-S-2A | 0.80 |
| 673 | **P26059B** | 0.01 |

^a^ Proportion among virome reads that were assigned to viruses

**Supplementary Table S4.** List of the most highly-assigned viruses in the competitive binning analysis of Lake Michigan virome (SRR1974488)

| Rank | Name | %^a^ |
| --- | --- | --- |
| 1 | *Pseudomonas* phage PB1 | 10.09 |
| 2 | *Puniceispirillum* phage HMO-2011 | 4.26 |
| 3 | *Pseudomonas* phage phiKZ | 2.86 |
| 4 | *Pelagibacter* phage HTVC008M | 2.21 |
| 5 | *Persicivirga* phage P12024S | 2.10 |
| 6 | *Pseudomonas* phage F8 | 1.70 |
| 7 | *Pseudomonas* phage DL60 | 1.61 |
| 8 | *Pelagibacter* phage HTVC010P | 1.40 |
| 9 | *Pectobacterium* phage phiTE | 1.37 |
| 10 | Yellowstone lake phycodnavirus 3 | 1.23 |
| 11 | LD28 phage, P19250A | 1.16 |
| 12 | *Chrysochromulina* *ericina* virus | 1.14 |
| 13 | *Prochlorococcus* phage P-TIM68 | 1.08 |
| 14 | *Pseudomonas* phage vB-Pae-PS44 | 1.01 |
| 15 | **P26059A** | 1.00 |
| 16 | *Pseudomonas* phage KPP12 | 1.00 |
| 17 | *Celeribacter* phage P12053L | 0.91 |
| 18 | *Pelagibacter* phage HTVC019P | 0.88 |
| 19 | *Phaeocystis* *globose* virus | 0.84 |
| 20 | *Acanthocystis turfaceae Chlorella* virus 1 | 0.80 |
| 67 | **P26059B** | 0.31 |

^a^ Proportion among virome reads that were assigned to viruses

**Supplementary Table S5.** List of the most highly-assigned viruses in the competitive binning analysis of Lake Michigan virome (SRR1974494)

| Rank | Name | %^a^ |
| --- | --- | --- |
| 1 | *Persicivirga* phage P12024S | 6.55 |
| 2 | *Puniceispirillum* phage HMO-2011 | 5.81 |
| 3 | *Pelagibacter* phage HTVC008M | 4.31 |
| 4 | *Pseudomonas* phage PB1 | 3.30 |
| 5 | LD28 phage, P19250A | 2.21 |
| 6 | **P26059A** | 2.20 |
| 7 | *Persicivirga* phage P12024L | 2.08 |
| 8 | *Synechococcus* phage S-SM2 | 2.00 |
| 9 | Yellowstone lake phycodnavirus 3 | 1.81 |
| 10 | *Cronobacter* phage vB CsaM GAP32 | 1.59 |
| 11 | *Prochlorococcus* phage P-TIM68 | 1.51 |
| 12 | *Pelagibacter* phage HTVC010P | 1.22 |
| 13 | *Synechococcus* phage S-CBS2 | 1.16 |
| 14 | Yellowstone lake phycodnavirus 2 | 1.14 |
| 15 | *Synechococcus* phage S-SKS1 | 1.08 |
| 16 | Cyanophage KBS-S-2A | 0.95 |
| 17 | *Celeribacter* phage P12053L | 0.95 |
| 18 | *Synechococcus* phage S-SSM7 | 0.92 |
| 19 | *Caulobacter* phage Cr30 | 0.90 |
| 20 | *Prochlorococcus* phage P-SSM2 | 0.87 |
| 160 | **P26059B** | 0.11 |

^a^ Proportion among virome reads that were assigned to viruses

**Supplementary Table S6.** List of the most highly-assigned viruses in the competitive binning analysis of Lake Michigan virome (SRR1974497)

| Rank | Name | %^a^ |
| --- | --- | --- |
| 1 | *Flavobacterium* phage 11b | 7.77 |
| 2 | *Cellulophaga* phage phi38:1 | 6.30 |
| 3 | *Puniceispirillum* phage HMO-2011 | 4.57 |
| 4 | *Persicivirga* phage P12024S | 3.66 |
| 5 | *Pelagibacter* phage HTVC008M | 2.62 |
| 6 | *Pseudomonas* phage PB1 | 1.80 |
| 7 | **P26059B** | 1.55 |
| 8 | Yellowstone lake phycodnavirus 3 | 1.51 |
| 9 | *Rhodoferax* phage P26218 | 1.42 |
| 10 | LD28 phage, P19250A | 1.42 |
| 11 | *Cronobacter* phage vB CsaM GAP32 | 1.12 |
| 12 | *Synechococcus* phage S-SM2 | 1.02 |
| 13 | *Persicivirga* phage P12024L | 0.98 |
| 14 | *Synechococcus* phage S-SKS1 | 0.96 |
| 15 | *Prochlorococcus* phage P-TIM68 | 0.95 |
| 16 | Yellowstone lake phycodnavirus 2 | 0.91 |
| 17 | *Pelagibacter* phage HTVC010P | 0.86 |
| 18 | *Bordetella* virus BPP1 | 0.85 |
| 19 | *Celeribacter* phage P12053L | 0.83 |
| 20 | *Chrysochromulina ericina* virus | 0.78 |
| 21 | **P26059A** | 0.77 |

^a^ Proportion among virome reads that were assigned to viruses

**Supplementary Table S7.** List of the most highly-assigned viruses in the competitive binning analysis of Lake Michigan virome (SRR1974501)

| Rank | Name | %^a^ |
| --- | --- | --- |
| 1 | *Puniceispirillum* phage HMO-2011 | 7.32 |
| 2 | *Chrysochromulina* ericina virus | 3.21 |
| 3 | *Pelagibacter* phage HTVC008M | 3.03 |
| 4 | *Persicivirga* phage P12024S | 2.65 |
| 5 | *Flavobacterium* phage 11b | 2.56 |
| 6 | Yellowstone lake phycodnavirus 3 | 2.34 |
| 7 | *Phaeocystis* *globosa* virus | 1.73 |
| 8 | Yellowstone lake phycodnavirus 2 | 1.60 |
| 9 | LD28 phage, P19250A | 1.57 |
| 10 | *Cronobacter* phage vB CsaM GAP32 | 1.47 |
| 11 | *Pseudomonas* phage PB1 | 1.31 |
| 12 | *Celeribacter* phage P12053L | 1.29 |
| 13 | **P26059B** | 1.21 |
| 14 | *Pelagibacter* phage HTVC010P | 1.20 |
| 15 | Yellowstone lake phycodnavirus 1 | 1.18 |
| 16 | *Synechococcus* phage S-SKS1 | 1.09 |
| 17 | *Synechococcus* phage S-SM2 | 1.07 |
| 18 | *Prochlorococcus* phage P-TIM68 | 1.06 |
| 19 | Yellowstone lake mimivirus | 0.91 |
| 20 | *Persicivirga* phage P12024L | 0.78 |
| 30 | **P26059A** | 0.64 |

^a^ Proportion among virome reads that were assigned to viruses

**Supplementary Table S8.** List of the most highly-assigned viruses in the competitive binning analysis of Lake Michigan virome (SRR1974511)

| Rank | Name | %^a^ |
| --- | --- | --- |
| 1 | *Puniceispirillum* phage HMO-2011 | 5.75 |
| 2 | *Pelagibacter* phage HTVC008M | 4.65 |
| 3 | *Pseudomonas* phage PB1 | 4.00 |
| 4 | Yellowstone lake phycodnavirus 3 | 1.88 |
| 5 | *Cronobacter* phage vB CsaM GAP32 | 1.81 |
| 6 | Yellowstone lake phycodnavirus 2 | 1.33 |
| 7 | *Chrysochromulina* *ericina* virus | 1.30 |
| 8 | *Pseudomonas* phage phiKZ | 1.30 |
| 9 | LD28 phage, P19250A | 1.23 |
| 10 | *Pelagibacter* phage HTVC010P | 1.16 |
| 11 | *Idiomarinaceae* phage 1N2-2 | 1.11 |
| 12 | *Synechococcus* phage S-SM2 | 1.10 |
| 13 | *Salicola* phage CGphi29 | 1.10 |
| 14 | *Prochlorococcus* phage P-TIM68 | 1.06 |
| 15 | *Prochlorococcus* phage P-SSM2 | 1.05 |
| 16 | **P26059A** | 1.01 |
| 17 | *Phaeocystis globosa* virus | 0.90 |
| 18 | *Celeribacter* phage P12053L | 0.90 |
| 19 | Yellowstone lake phycodnavirus 1 | 0.89 |
| 20 | *Caulobacter* phage Cr30 | 0.89 |
| 281 | **P26059B** | 0.05 |

^a^ Proportion among virome reads that were assigned to viruses

**Supplementary Table S9.** List of the most highly-assigned viruses in the competitive binning analysis of Lake Michigan virome (SRR1974513)

| Rank | Name | %^a^ |
| --- | --- | --- |
| 1 | *Puniceispirillum* phage HMO-2011 | 6.19 |
| 2 | *Pseudomonas* phage PB1 | 4.26 |
| 3 | *Pelagibacter* phage HTVC008M | 3.09 |
| 4 | *Burkholderia* phage BcepNazgul | 2.25 |
| 5 | *Burkholderia* phage AH2 | 1.99 |
| 6 | *Persicivirga* phage P12024S | 1.46 |
| 7 | LD28 phage, P19250A | 1.34 |
| 8 | **P26059A** | 1.27 |
| 9 | *Pseudomonas* phage phiKZ | 1.17 |
| 10 | *Pelagibacter* phage HTVC010P | 1.09 |
| 11 | *Cronobacter* phage vB CsaM GAP32 | 1.08 |
| 12 | *Prochlorococcus* phage P-TIM68 | 1.05 |
| 13 | Yellowstone lake phycodnavirus 3 | 1.04 |
| 14 | *Synechococcus* phage S-SM2 | 0.98 |
| 15 | *Caulobacter* phage Cr30 | 0.92 |
| 16 | Enterobacter phage Enc34 | 0.90 |
| 17 | *Cellulophaga* phage phi14:2 | 0.84 |
| 18 | *Proteus* phage pPM 01 | 0.80 |
| 19 | *Flavobacterium* phage 11b | 0.79 |
| 20 | *Synechococcus* phage S-SKS1 | 0.77 |
| 126 | **P26059B** | 0.19 |

^a^ Proportion among virome reads that were assigned to viruses
